# Editorial: MALDI-TOF MS Application in Microbial Ecology Studies

**DOI:** 10.3389/fmicb.2019.02954

**Published:** 2020-01-10

**Authors:** Praveen Rahi, Parag Vaishampayan

**Affiliations:** ^1^National Centre for Microbial Resource, National Centre for Cell Science, Pune, India; ^2^Biotechnology and Planetary Protection Group, Jet Propulsion Laboratory, California Institute of Technology, Pasadena, CA, United States

**Keywords:** high-throughput (HT) approaches, culturomics, microbial biological resource centers, antimicrobial resistance (AMR), strain typing, dereplication

Recent advancements in high-throughput sequencing, proteomics, metabolomics, and bioinformatics have revolutionized the microbial ecology research and immensely improved our understanding of the microbiome. Metagenomics and other cultivation-independent studies have shown that a diverse population of hundreds of millions of microorganisms habitat various ecosystems of the earth. The majority of these isolates have not yet been cultivated, and their metabolic functions remain unknown. Cultivation of microorganisms holds several essential advantages, which includes the prospection of isolated culture for their potential biotechnological applications. Though it is impossible to cultivate all the microbial community members, cultivation of majority of gut bacterial community members, including several novel taxa has been achieved by using multiple culture conditions approach (Lagier et al., 2016).

Such studies involve simulation of culture conditions by mimicking the natural environment and high-throughput cultivation of microorganisms, leading to the development of a new approach known as “Culturomics” (Lagier et al., 2012). A rapid and reliable method for the identification of microorganisms from various ecosystems is critically required to meet the requirements of culturomics. Matrix-assisted laser desorption/ionization time-of-flight mass spectrometry (MALDI-TOF MS), based identification of microorganisms, appears the most suitable technique for this role. The ability to generate “portable” data to develop databases holds the key to its successful application in microbial ecology studies. In addition to high-throughput identification, MALDI-TOF MS has also been used for the analysis of microbial function or metabolism at the single organism and/or whole community level by the profiling of various biomolecules like proteins, sugar, and lipids (Clark et al., 2018; Weigt et al., 2018).

The articles published under this Research Topic reported application of MALDI-TOF MS for the accurate and fast identification of microorganisms isolated from diverse environments including nosocomial settings (Florio et al.), biofilm habitats (Tuohy et al.), spacecraft and associated surfaces (Seuylemezian et al.), and cellular phones (Kurli et al.). In addition to this, a minireview highlighted the advantages and challenges associated with MALDI-TOF MS based microbial diagnosis and identification (Florio et al.), and an opinion article pointed to toward the collaborative efforts toward curated database especially for fungal community ecology (Lima et al.). In this editorial, we did a critical analysis of each article published under the Research Topic, and propose various solutions to improve the application of MALDI-TOF MS in microbial ecology studies.

## Recent Advances in MALDI-TOF MS in Microbial Identification

MALDI-TOF MS is widely used in routine identification of microbial pathogens and has influenced clinical diagnostics and is replacing existing identification methods, including both biochemical and 16S rRNA gene sequencing. The rapid identification of microorganisms causing nosocomial infections, in addition to the knowledge of the antibiotic resistance patterns is highly advantageous especially in terms of morbidity, mortality, and health costs savings (Florio et al.). New perspectives are being explored for MALDI-TOF MS in microbial identification, such as analyzing direct positive blood cultures for identification of pathogens, sub-species and strain typing, detection of drug resistance determinants, and generating specialized metabolite MS profiles to assess bacterial functional traits (Florio et al.; Clark et al., 2018; Oviaño and Bou, 2018). Most of these applications of MALDI-TOF MS are in the preliminary stage of development and require more standardization to provide reliable solutions. The applications of MALDI-TOF MS in the rapid identification of bacteria from positive blood cultures, which allow identification of infection-causing bacteria instantly (Florio et al.) The impact of sample processing methods on the observed variability incorrect identification rates was discussed in this mini-review, but authors missed to give any recommendations to improve the identifications from positive blood culture. Recently, a small modification in the sample processing allowed identification of *Brucella* infection directly from positive blood culture with MALDI-TOF MS (Bruker) within 2 h, and saved almost 48 h from positive blood culture to correct identification of pathogen (Guo et al., 2019). The development of MALDI-TOF MS based methods for the assessment of antimicrobial resistance in both bacteria and fungi are also in the early stage of development (Florio et al.) More focused studies on the development and validation of new methods will be necessary before MALDI-TOF MS can be used in routine assays for the diagnosis of antimicrobial resistance.

Recently, a MALDI-TOF mass spectrometry data acquisition and bioinformatics pipeline (IDBac) is designed by integrating the data from both intact protein and specialized metabolite spectra directly from bacterial cells grown on agar (Clark et al., 2018). This coupling of MALDI-TOF mass spectrometry protein and specialized metabolite analyses resulted in establishing an instant connection between microbial phylogenetic identities with potential environmental functions. Such innovations allow utilization of full capabilities offered by MALDI-TOF MS for the analyses of microbial metabolites and the identification of microorganisms (Clark et al., 2018). MALDI-TOF MS is also finding applications in removing the redundancy, in big microbial discovery and bioprospection projects. MALDI-TOF MS and the bioinformatics pipeline IDBac offers a cost-effective way to rapidly de-replicate the microbial collection based on both the bacterial identity and the natural product producing capacity of colonies in high-throughput (Costa et al., 2019).

MALDI-TOF MS based identification becoming a must for the large-scale culturomics experiments, as it is a fast and cost-effective method to filter out multiple conspecific strains or genetically identical clones (Dumolin et al., 2019). Though, the limited database and bias of commercial databases toward microbes of clinical or food safety relevance restrict the application of MALDI-TOF MS. Methods based on similarity measures combined with the clustering of MALDI-TOF mass spectra have been developed to uncouple dereplication from database based identification. Till recently, the identification of identical strains was done by visual interpretation of dendrograms or by using a distance cutoff value (Strejcek et al.). Recently, an algorithm (SPeDE) has been introduced, which enables the rapid analysis and dereplication of isolates using comparison of MALDI-TOF MS profiles (Dumolin et al., 2019). This approach identifies unique spectral features between pairs of spectra and clusters them into operational isolation units (OIUs).

MALDI-TOF MS based clustering has also been used to reveal a potential clonal route of transmission of *Enterobacter* spp. preferentially between general surgery and geriatric wards, which were different from strains of other wards (Florio et al.). This study represents an important source of information about the spreading of *Enterobacter*, which is an emergent pathogen for its ability to acquire determinants of antimicrobial resistance and role of MALDI-TOF MS in microbiological surveillance in nosocomial setting (Florio et al.). In addition to microorganisms of clinical origin, MALDI-TOF MS offered a high-resolution approach to differentiate environmental isolates of the genus *Deinococcus* at the subpopulation level, isolated from a freshwater system from other environmental niches (Tuohy et al.). Though the sample size was small (19 isolates) in the study, still it was able to demonstrate the capability of MALDI-TOF MS as a reliable tool to differentiate *Deinococcus aquaticus* isolates that originate from specific niches (Tuohy et al.). Use of MALDI-TOF MS data for efficient dereplication of recurrent bacterial isolates for downstream analyses was suggested with minimal loss of unique organisms (Strejcek et al.).

## Challenges in MALDI-TOF MS in Microbial Identification

Several studies concluded that low reliable identification of microbes from non-clinical ecosystems such as soil, water, spacecraft assembly cleanrooms, is due to the heavy dominance of MS profiles from clinical isolates in the database (Rahi et al., 2016; Seuylemezian et al.; Strejcek et al.). MALDI-TOF MS based microbial identification platforms allow creating and including a custom, user-specific, and transferable database (Rau et al., 2016; Seuylemezian et al.). The development of a custom database of MALDI-TOF MS profiles of bacterial isolates obtained from spacecraft and associated cleanroom environments has led to the successful identification of 454 bacterial isolates in concurrence with their 16S rRNA gene sequence-based classifications (Seuylemezian et al.). In this study, authors also observed that MALDI-TOF MS could resolve strain-level variations, identified potential novel species and distinguished between members of taxonomic groups, which is not possible using conventional 16S rRNA gene sequencing (Seuylemezian et al.). The results of both 16S rRNA gene sequence-based EzBioCloud Identify service (REF/site) and the MALDI Biotyper database corroborated well in identifying the bacterial strains up to the genus-level, except four unreliably identification by the MALDI Biotyper method (Strejcek et al.). The study also highlighted that only ~35% species-level identifications by MALDI Biotyper could coincide with those identified by 16S rRNA gene sequencing analysis, indicating weak species-level identification by MALDI-TOF MS (Strejcek et al.).

Insufficient coverage of bacterial species in the databases has been suggested as the primary reason for these discrepancies (Strejcek et al.). Lack of public repository to submit the new spectral references generated by researchers further worsen this problem (Seuylemezian et al.; Strejcek et al.). The problem worsens in the absence of clear standards and tools to verify the accuracy of the reference profiles. However, the attempts are being made to define a universal MALDI spectral database (Rahi et al., 2016). Simultaneous, efforts have been made to develop user-to-user internet platforms like MALDI-UP specifically designed to facilitate the exchange of in-house MALDI spectral database entries (Rau et al., 2016). In addition to this, researchers might be sharing the spectral database formally and informally, and such exercise will help to develop a consensus on the universal codes for the high-quality spectral entries.

All the database repository efforts and online user-to-user platforms have hidden challenges of erroneous database entries, the requirement of continuous curation and validation. These challenges could be the bottleneck to spectral data sharing and repository. The deposition of microbial strains with Microbial Biological Resource Centers (mBRCs) to make them accessible to the research community for verifications and validations of the MALDI-TOF MS spectral database could be a solution to this problem. Although the biodiversity laws of several countries do not allow free movement of microbial cultures, hindering such international efforts (Soltanighias et al., 2018). Blind classification of the microbial strains and inter-lab comparisons could help validate the newly generated spectral profiles.

The availability of freely accessible, well-curated MALDI-TOF MS spectra database that allows query search using a web-based interface, comparable to BLAST search on the NCBI GenBank website and databases, could help researchers to identify microbial isolates, without adding spectral profiles into their in-house database (Normand et al., 2017). In a collaborative effort, researchers from five laboratories in France and Belgium developed a free access software to scan a fungal database, which includes spectra of strains belonging to 938 species and 246 genera (Normand et al., 2017). Although at present this tool is limited to the identification of fungi, similar tools can be developed for other microbial groups, and here the mBRCs, need to play a significant proactive role as they have extensive collections of well-curated microbial strains.

The quality and accuracy of MALDI-TOF MS spectra can be influenced by the sample preparation methods, matrix components, and type of material analyzed (Lima et al.). During the identification of an extensive collection of microorganisms isolated from cellular phone surfaces, it was observed that quality filtering of MALDI-TOF MS spectrum, improving sample processing methods and enriching the spectral database lead to better and highly reliable results for microbial identifications by MALDI-TOF MS (Kurli et al.). In this study, genus-level identity was achieved for nearly 80% isolates (Kurli et al.). Improvements in the quality of MALDI-TOF MS spectrum (i.e., peak number and intensity) can reduce the number of no reliable identification and also lower the number of erroneous identification (Kurli et al.). If we could find feasible solutions for the challenges associated with MALDI-TOF MS, this technique has the potential to become a “golden standard” for the identification of diverse groups of bacteria and fungi (Lima et al.).

The development of newer algorithms and sample processing methods will improve the applications of MALDI-TOF MS and increase its relevance in microbial ecology studies. Further standardization and ease of accessibility of the mass spectral libraries from variety of environmental sources, and not just clinical origin, will make it the method of choice for microbial ecology studies. Application of the MALDI-TOF MS in large-scale screening and identification studies will increase in the near future, due to the advances in the automation and de-replication, and lead to the discovery of novel microbial species.

## Author Contributions

All authors listed have made a substantial, direct and intellectual contribution to the work, and approved it for publication.

### Conflict of Interest

The authors declare that the research was conducted in the absence of any commercial or financial relationships that could be construed as a potential conflict of interest.

## References

[B1] Clark C. M. Costa M. S. Sanchez L. M. Murphy B. T. (2018). Coupling MALDI-TOF mass spectrometry protein and specialized metabolite analyses to rapidly discriminate bacterial function. Proc. Natl. Acad. Sci. U.S.A. 115, 4981–4986. 10.1073/pnas.1801247115 29686101 PMC5949002

[B2] Costa M. S. Clark C. M. Ómarsdóttir S. Sanchez L. M. Murphy B. T. (2019). Minimizing taxonomic and natural product redundancy in microbial libraries using MALDI-TOF MS and the bioinformatics pipeline IDBac. J. Nat. Prod. 82, 2167–2173. 10.1021/acs.jnatprod.9b00168 31335140 PMC7197193

[B3] Dumolin C. Aerts M. Verheyde B. Schellaert S. Vandamme T. Van der Jeugt F.. (2019). Introducing SPeDE: high-throughput dereplication and accurate determination of microbial diversity from matrix-assisted laser desorption-ionization time of flight mass spectrometry data. mSystems 4:e00437–19. 10.1128/mSystems.00437-19 31506264 PMC6739102

[B4] Guo J. Lai W. Li B. Tang L. Wu Y. Luo Y.. (2019). Rapid identification of Brucella sepsis/osteomyelitis in a 6-year old febrile patient with matrix-assisted laser desorption/ionization time-of-flight mass spectrometry directly from positive blood culture: a case report. BMC Infect. Dis. 19:240. 10.1186/s12879-019-3864-z 30871483 PMC6419399

[B5] Lagier J.-C. Khelaifia S. Alou M. T. Ndongo S. Dione N. Hugon P.. (2016). Culture of previously uncultured members of the human gut microbiota by culturomics. Nat. Microbiol. 1:16203. 10.1038/nmicrobiol.2016.203 27819657 PMC12094094

[B6] Lagier J. C. Armougom F. Million M. Hugon P. Pagnier I. Robert C.. (2012). Microbial culturomics: paradigm shift in the human gut microbiome study. Clin. Microbiol. Infect. 18, 1185–1193. 10.1111/1469-0691.12023 23033984

[B7] Normand A. C. Becker P. Gabriel F. Cassagne C. Accoceberry I. Gari-Toussaint M.. (2017). Validation of a new web application for identification of fungi by use of matrix-assisted laser desorption ionization-time of flight mass spectrometry. J. Clin. Microbiol. 55, 2661–2670. 10.1128/JCM.00263-17 28637907 PMC5648703

[B8] Oviaño M. Bou G. (2018). Matrix-assisted laser desorption ionization–time of flight mass spectrometry for the rapid detection of antimicrobial resistance mechanisms and beyond. Clin. Microbiol. Rev. 32:e00037–e00018. 10.1128/CMR.00037-18 30487165 PMC6302359

[B9] Rahi P. Prakash O. Shouche Y. S. (2016). Matrix-assisted laser desorption/ionization time-of-flight mass-spectrometry (MALDI-TOF MS) based microbial identifications: challenges and scopes for microbial ecologists. Front. Microbiol. 7:1359. 10.3389/fmicb.2016.01359 27625644 PMC5003876

[B10] Rau J. Eisenberg T. Wind C. Lasch P. Sting R. (2016). MALDI-UP – An internet platform for the exchange of MALDI-TOF mass spectra. Asp. Food Control Anim. Heal. J. 1, 1–17. Available online at: http://maldi-up.ua-bw.de/

[B11] Soltanighias T. Vaid R. K. Rahi P. (2018). Agricultural Microbial Genetic Resources: Application and Preservation at Microbial Resource Centers, in Microbial Resource Conservation, eds Sharma S. K. Verma A. (Cham: Springer), 141–173. 10.1007/978-3-319-96971-8_5

[B12] Weigt D. Sammour D. A. Ulrich T. Munteanu B. Hopf C. (2018). Automated analysis of lipid drug-response markers by combined fast and high-resolution whole cell MALDI mass spectrometry biotyping. Sci. Rep. 8:1120. 10.1038/s41598-018-29677-z 30050068 PMC6062520

